# Effect of Early Continuous Veno-Venous Haemofiltration in Severe Acute Pancreatitis for the Prevention of Local Pancreatic Complications

**DOI:** 10.1155/2022/7575231

**Published:** 2022-03-07

**Authors:** Satish Chandra Yadav, Bingqiang Zhang

**Affiliations:** The First Affiliated Hospital Chongqing Medical University, China

## Abstract

**Objective:**

To compare the conventional treatment and continuous veno-venous haemofiltration (CVVH) in severe acute pancreatitis (SAP) for the prevention of pseudocyst and walled-off necrosis. *Patients and Methods*. Forty-two patients were divided into two treatment groups: conventional treatment group contained 24 patients and CVVH had 18. Conventional treatment group patients were treated symptomatically and according to the causes. CVVH group patients were treated symptomatically, and CVVH was done within 2 hours of admission.

**Results:**

In both groups, there was a decrease in amylase, lipase, CRP, IL-6, IL-10, TNF-alpha, Ranson score, Balthazar score, and APACHE-II score after 72 hours, but the decrease was significantly greater in CVVH patients. There were no any local pancreatic complications in CVVH patients, but 1 patient had an acute peripancreatic fluid collection, 2 patients had pseudocyst, and 2 patients had walled-off necrosis (WON), and a mortality one was seen in the conventional treatment group.

**Conclusion:**

The present study shows that early CVVH may be able to prevent the formation of pseudocyst and win in SAP patients.

## 1. Introduction

**Figure 1:**
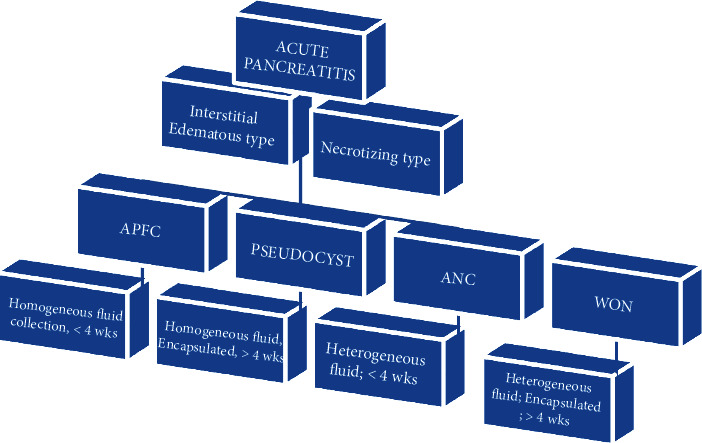
Revised Atlanta classification system of acute pancreatitis.

Acute pancreatitis (AP) is a sudden but reversible inflammatory process of the pancreas. AP is the leading GI aetiology of hospitalization in the USA [1]. The majority of pancreatitis cases in the Western countries are due to alcohol, and the majority of pancreatitis cases in the eastern countries are due to gallstone. Approximately 80% patients have mild pancreatitis, and 20% of patients have severe pancreatitis. About 15-25% of acute pancreatitis develops into severe pancreatitis [2]. The severity of pancreatitis has been redefined by the revised Atlanta classification system in 2012 [3] by international consensus, which classifies acute pancreatitis in mild, moderate, and severe types (Figure 1).

Acute pancreatitis without organ failure and local or systemic complications are called mild pancreatitis. Acute pancreatitis with transient organ failure which resolves in less than 48 hours and/or local or systemic complications without persistent organ failure, not lasting more than 48 hours, is called moderately severe acute pancreatitis. Acute pancreatitis with persistent one or more organ failure is called severe acute pancreatitis (SAP). SAP is a fatal ailment, which causes severe local as well as systemic complications. The local complication includes acute peripancreatic fluid collection (APFC), pancreatic pseudocyst, acute necrotic collection (ANC), and walled-off necrosis (WON). The systemic complications are sepsis, SIRS, and MODS. These complications of SAP have a high death rate [4].

The pancreatic pseudocyst is seen in 10–20% cases of acute pancreatitis. Most of the pseudocysts resolve spontaneously; however, those remaining cases of pseudocyst which do not resolve itself cause severe complications such as bleeding, gastric outlet or biliary obstruction, sepsis, haemorrhage, pseudoaneurysm formation, rupture, fistula, and WON [5]. These complications cause higher death rates. The invasive treatment of pseudocysts is percutaneous or endoscopic drainage and surgery [6, 7]. Percutaneous drainage has high morbidity and mortality [8]. Surgery is expensive and has many complications, so it is better to prevent pseudocyst formation in SAP cases.

Continuous veno-venous haemofiltration (CVVH) is a renal replacement therapy which is used in a critical care setting for maintaining cardiopulmonary function, for renal support, and for removal from the blood of inflammatory cytokines. CVVH has been used for SAP nearly two decades since 1991 [9]. In the most recent times, the use of CVVH has increased. The early CVVH can effectively clear cytokines and increase oxygen supply to the tissue and restore the microcirculation of the tissues. Thus, it can prevent the injury to the pancreatic parenchyma and translocation of gut bacteria. In this way, CVVH can prevent the formation of the pseudocyst.

In the present study, we are going to compare the CVVH and conventional treatment in the management of acute systemic complications as well as preventing the chronic local complications and mortality rate in SAP patients.

## 2. Patients and Methods

This study is a single-center retrospective study of Chongqing Medical University First Affiliated Hospital which is affirmed by its institutional review board. Prior to the study, consent was taken from the patients themselves or from their relatives. Forty-two patients who were admitted to the Chongqing Medical University First Affiliated Hospital, from September 15, 2014, to December 20, 2016, are included in this study. AP was diagnosed by the following 2 or more characteristics [10, 11]:

- Severe acute epigastric abdominal pain often radiates to the back
- Serum lipase or amylase is 3 times or more than the upper limit of normal value
- Characteristic findings of acute pancreatitis on imaging

SAP was diagnosed based on the revised Atlanta classification system (2012) [3], and organ failure was diagnosed according to the modified Marshall scoring system [12] as shown in (Table 1); the inclusion criteria were age more than 18 years, admission within 24 hours of onset, Ranson score of 3 or more, Acute Physiology and Chronic Health Evaluation II (APACHE II) score of 8 or more, and Balthazar score of 4 or more. The exclusion criteria were pregnancy, malignancy, immunodeficiency, and preexisting CKD requiring regular hemodialysis.

Among the 42 patients, 30 patients were male and 12 patients were female, aged between 31 and 83 years. In the CVVH group, the aetiology of SAP was alcohol (3 patients), biliary disease (12 patients), and hyperlipidemia (3 patients). The aetiology of SAP in the conventional group was alcohol (4 patients), biliary disease (15 patients), and hyperlipidemia (5 patients). Forty-two patients were divided into 2 groups: continuous veno-venous haemofiltration (CVVH, *N* = 18) or conventional recommended treatments (*N* = 24) according to the treatment model. Among the 42 patients, 18 patients (13 males and 5 females), aged 51.77 ± 12.02 years, were given continuous veno-venous haemofiltration (CVVH) along with supportive treatment and 24 patients (17 males and 7 females), aged 53.50 ± 12.89 years, were given recommended conventional treatments only.

### 2.1. Conventional Treatment

All 42 patients were admitted to an intensive care unit. Among them, 24 patients were given only the following recommended conventional treatments: (1) Nil per OS (NPO); (2) fluid resuscitation: normal saline or Ringer lactate; (3) oxygen supplementation: saturation maintained about 95%; (4) pain management: intravenous opiates, mainly morphine and fentanyl; (5) nutritional supports: NG tube feeding within 24 hours, which also decreases the risk of infection; (6) gastrointestinal decompression; (7) prophylactic antibiotics: carbapenems mainly Imipenem (7-10 days); and (8) management of underlying causes. In addition to the standard conventional treatment, patients in the CVVH group were also given CVVH at least for 72 hours.

## 3. CVVH Technique

Haemofiltration was done with Gambro Prisma flex machine (Gambro Lundia AB, hemp filters, Sweden) within two hours of hospital admission. The Seldinger method was used to insert the dual lumen central venous catheter (18 cm, ARROW, USA) percutaneously mostly into the internal jugular vein and few into the femoral vein. A polyacrylonitrile AN69-ST hemofilter with surface area of 1.2 m^2^ and weight limit of 35 kD (Hospal, USA) was used for haemofiltration. Blood and substitute fluid were infused at the 200-250 mL/min and 60-80 mL/kg per hour, respectively. Substitute fluids were added before and after the filtration. Low molecular weight heparins (Fragmin: 5000/ampule) were administered at a dose of 100-140 IU/kg, and bolus injection was made before haemofiltration. It was performed at a rate of 35 mL/kg per hour. The replacement solution was composed of 2000 mL normal saline, 5% NaHCO_3_ 125 mL, 25% MgSO4 solution 1 mL, 10% C_12_H_22_CaO_14_ 10 mL, and 5% glucose solution 500 mL. CVVH was continued for 72 hours, and a polyacrylonitrile AN69-ST hemofilter was changed in every 24 hours if there was no any complication in between.

### 3.1. Parameters Monitored and Biomarker Measurement

Vital signs, including blood pressure, temperature, heart rate, and respiration rate were monitored every half an hour. A blood sample was collected before and after the treatment to observe amylase, lipase, CRP, IL-6, IL-10, TNF-alpha, Ranson score, and APACHE-II score. Plasma levels of inflammatory cytokines, IL-6, IL-10, and TNF-alpha were measured before CVVH (at the time of admission) and after CVVH using ELISA kits (Siemens Healthcare Diagnostics IMMULITE 1000 USA machine) at 72 hours.

## 4. Statistical Analysis

The software SPSS version 20 (SPSS Inc., Chicago, IL) was used for the statistical analysis. Data analysis was done using Student's *t*-test. Results were expressed as the mean ± standard deviation (SD). The two-tailed *p* value (*p* < 0.05) was considered statistically significant. The bar chart was made using Excel version 2016.

## 5. Results

### 5.1. Change in Biochemistry Parameters: Amylase, Lipase, and CRP

The amylase (635.55 ± 138.50), lipase (1067.22 ± 153.19), and CRP (269.27 ± 75.77) in the CVVH group were not significantly different from the conventional group's amylase (632.95 ± 145.14), lipase (1066.91 ± 124.34), and CRP (268.79 ± 48.50) at the time of admission, and *p* > 0.05 was not statistically significant as shown in (Table 2).

The pretreatment amylase (632.95 ± 145.14), lipase (1066.91 ± 124.34), and CRP (268.79 ± 48.50) in the conventional group were significantly different from posttreatment amylase (507.87 ± 99.87), lipase (939.37 ± 118.01), and CRP (226.50 ± 39.89), and *p* < 0.01 was statistically significant as shown in Table 3.

The pretreatment amylase (635.55 ± 138.50), lipase (1067.22 ± 153.19), and CRP (269.27 ± 75.77) in the CVVH group were significantly different from posttreatment amylase (242.22 ± 58.85), lipase (430.27 ± 98.30), and CRP (159.27 ± 16.06), and *p* < 0.01 was statistically significant as shown in Table 4.

### 5.2. Change in Severity Score: Ranson Score, APACHE-II Score, and Balthazar Score

The Ranson score (6.00 ± 1.45), APACHE-II score (21.83 ± 1.75), and Balthazar score (7.27 ± 0.75) in the CVVH group were statistically alike to the conventional group's Ranson score (5.79 ± 1.50), APACHE-II score (22.66 ± 1.78), and Balthazar score (6.95 ± 0.75) at the time of admission, and *p* > 0.05 was not statistically significant as shown in Table 2.

The pretreatment Ranson score (5.79 ± 1.50) and APACHE-II score (22.66 ± 1.78) in the conventional group were significantly different from posttreatment Ranson score (5.00 ± 1.17) and APACHE-II score (19.45 ± 1.31) (*p* < 0.05), but pretreatment Balthazar score (6.95 ± 0.75) vs. posttreatment Balthazar score (6.62 ± 0.64) was not statistically significant (*p* > 0.05) (Table 3).

The pretreatment Ranson score (6.00 ± 1.45), APACHE-II score (21.83 ± 1.75), and Balthazar score (7.27 ± 0.75) in the CVVH group were significantly different from posttreatment Ranson score (3.72 ± 0.75), APACHE-II score (11.27 ± 2.05), and Balthazar score (3.83 ± 0.85), and *p* < 0.01 was statistically significant as shown in Table 4.

### 5.3. Change in Cytokine Level: IL-6, IL-10, and TNF-Alpha

The IL-6 (662.22 ± 166.43), IL-10 (1104.05 ± 141.03), and TNF-alpha (577.94 ± 104.03) in the CVVH group were similar to the conventional treatment group's IL-6 (663.25 ± 167.32), IL-10 (1105.58 ± 107.14), and TNF-alpha (578.95 ± 82.95) before treatment, and *p* > 0.05 was not statistically significant as shown in Table 2.

The pretreatment IL-6 (663.25 ± 167.32), IL-10 (1105.58 ± 107.14), and TNF-alpha (578.95 ± 82.95) in the conventional group were significantly different from posttreatment IL-6 (570.12 ± 91.03), IL-10 (1024.62 ± 121.87), and TNF-alpha (517.00 ± 80.05), and *p* < 0.05 was statistically significant as shown in Table 3.

The pretreatment IL-6 (662.22 ± 166.43), IL-10 (1104.05 ± 141.03), and TNF-alpha (577.94 ± 104.03) in the CVVH group were significantly different from posttreatment IL-6 (274.27 ± 66.34), IL-10 (531.16 ± 76.33), and TNF-alpha (307.16 ± 58.78), and *p* < 0.01 was statistically significant as shown in Table 4.

**Figure 2:**
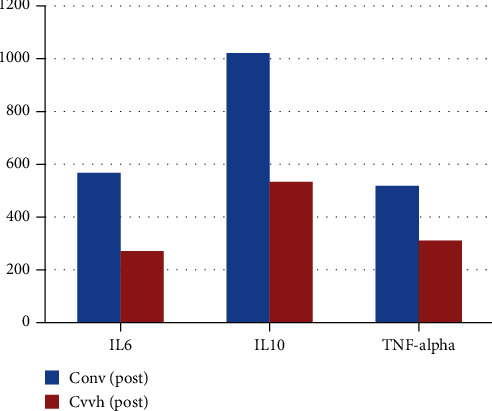
Bar chart: posttreatment IL-6, IL-10, and TNF-alpha in both groups; their difference was statistically significant (*p* < 0.01).

The posttreatment IL-6, IL-10, and TNF-alpha in the CVVH group were statistically significant compared to the conventional group (*p* < 0.01) (Figure 2).

### 5.4. Follow-Up and Complications

All 42 patients were followed from 1 month to 1 year with average 4.60 ± 2.91 months. The patients were followed up with history, physical examination, lab test, and ultrasound. If the symptoms and laboratory values or ultrasound indicated the need for CT, then a CT scan or MRI was done. In the CVVH group, there were no any local complications (acute peripancreatic fluid collection, pancreatic pseudocyst, acute necrotic collection, or walled-off necrosis) during follow-up. In the conventional therapy group, 1 patient had an acute peripancreatic fluid collection, 2 patients had pseudocyst, and 2 patients had walled-off necrosis and one death.

## 6. Discussion

There are 5 (3 cases, 2 comparisons) studies, which compared the cytokine level pre- and post-CVVH [13–17]. Results of the present study (CVVH group (0 h vs. 72 h): IL-6 (662.22 ± 166.43 vs. 274.27 ± 66.34), IL-10 (1104.05 ± 141.03 vs. 531.16 ± 76.33), and TNF-alpha (577.94 ± 104.03 vs. 307.16 ± 58.78)) are statistically significant (*p* < 0.01) and similar to the above studies' results. In the conventional treatment group (0 h vs. 72 h), IL-6 is 663.25 ± 167.32 vs. 570.12 ± 91.03, IL-10 is 1105.58 ± 107.14 vs. 1024.62 ± 121.87, and TNF-alpha is 578.95 ± 82.95 vs. 517.00 ± 80.05; although the level of IL-6, IL-10, and TNF-alpha decreased after the 72 hours of treatment and results were statistically significant (*p* < 0.05), the decrease was very small. This result shows that conventional treatment is not as effective as CVVH in clearing serum cytokines.

There are seven studies, which compared the severity index of pancreatitis at the time of admission and post-CVVH [13, 17–22]: Ranson score (6.00 ± 1.45 vs. 3.72 ± 0.75), APACHE-II score (21.83 ± 1.75 vs. 11.27 ± 2.05), and Balthazar score (7.27 ± 0.75 vs. 3.83 ± 0.85); their results are similar to the present study with statistical significance at *p* < 0.01. The conventional group's Ranson score (5.79 ± 1.50 vs. 5.00 ± 1.17) (*p* < 0.05) and APACHE-II score (22.66 ± 1.78 vs. 19.45 ± 1.31) (*p* < 0.01) were statistically significantly, but Balthazar score (6.95 ± 0.75 vs. 6.62 ± 0.64) was not statistically significant (*p* > 0.05). The present study shows that even though the Ranson score, APACHE-II score, and Balthazar score improved postconventional management, the improvement of the Balthazar score was small and not statistically significant and not as effective as CVVH.

There are five studies which compared the serum enzyme levels (pre- and post-CVVH) [13, 19, 20, 22, 23]. All four study results show the significant decrease in serum pancreatic enzyme levels post-CVVH, which is similar to the present study results. These enzymes were also decreased postconventional treatment, but the decrease was in a lesser proportion compared to CVVH. There are nine studies [14–18, 20, 21, 23, 24] that compared the mortality rate among CVVH patients; their result shows that the overall death rate is 6–50% and the death rate significantly decreases after CVVH. The present study also shows no death in the CVVH group but (1/24) 4.11% death rate in the conventional group.

During acute pancreatitis, trypsinogen comes in contact with the lysosomal enzyme cathepsin, which abnormally activates trypsinogen into trypsin inside the pancreas. Trypsin leads to the activation of other molecules of trypsinogen, and these enzymes together lead to inflammation, oedema, and vascular injury. During acute mild pancreatitis, there is a mild inflammation because very few inflammatory mediators are released. These inflammation and oedema subside in a few days time. However, in severe acute pancreatitis, there is extensive acinar cell injury, which leads to neutrophil, monocyte, and lymphocyte activation, and this results in the secretion of plenty of inflammatory mediators such as TNF-*α*, IL-6, and IL-10.

The proinflammatory and anti-inflammatory factors become unregulated and interact with each other, which causes the undue and long-lasting release of these cytokines. These inflammatory mediators (IL-6, TNF-*α*, IL-10, and nitric oxide) form a complex inflammatory network, which causes a cascade of chain reactions. These chain reactions cause endothelial damage, alter the microcirculation and cellular function, and decrease the immune response resulting in acute complications such as SIRS, MODS, ARDS, and chronic complications such as pseudocyst and WON.

A pancreatic pseudocyst is a delayed complication of acute pancreatitis (5–15%) or chronic pancreatitis (20–40%) and rarely pancreatic trauma [25, 26]. The formation of the pseudocyst takes more than 4 weeks to develop due to the maturation of acute inflammatory mediators, which causes injury to the parenchyma or ductal system of the pancreas, resulting in escaping of enzymes [3, 27]. WON is a result of trauma or secondary infection of the pancreatic pseudocyst [28]. Formation of WON takes four or more weeks from the onset of pancreatitis [29]. In the present study, no ERCP or any other traumatic procedure was done in either group which is also the cause of pseudocyst formation. The excessive positive water balance can induce peripancreatic fluid, pancreatic pseudocyst, and infection in SAP. In the present study, there was no positive water balance in the conventional treatment group; crystalloid fluid was given to maintain renal perfusion, targeting urine output at 0.5 mL/kg/h.

Aggressive conventional treatment can control SIRS and MODS in SAP patients in the acute setting in the few cases, but the chance of APFC, pseudocyst, ANC, and WON is still high since the enzymes and cytokines are not cleared timely and effectively with conventional treatment. The longer duration and higher amount of cytokines in the blood have a higher chance of pancreatic parenchymal damage, gut bacterial translocation, and duct disruption leading to higher risk of APFC, pseudocyst, ANC, and WON formation.

After the advance understanding of the path physiology of SAP, the treatment mode has changed over the past few years. The early and aggressive management of SAP has been used extensively, such as TNF-alpha monoclonal antibodies, IL receptor antagonist, PAF antagonist [30], persistence of surgery [31], peritoneal lavage [32], dialysis [19], continuous arterial infusion of protease inhibitor [33, 34], and endothelin receptor antagonist to reduce capillary leakage [35], in addition to intensive care. During SAP, both pro- and anti-inflammatory mediators become unregulated and interact with each other, leading to cascades of chain reactions, so neither antagonist treatment directed at one mediator nor the single dose intervention was effective. Thus, the results were not satisfactory and death rates were still high.

Currently, there is no any effective method, which can clear cytokines as efficiently as haemofiltration [9]. During CVVH, micromolecules (blood, urea nitrogen, creatinine, etc.) are removed by a diffusion process and medium-size substances such as cytokines are removed by the convection and adsorption process. The molecular weight of a substance is a key step in determining the capacity of a hemofilter to clear it [36]. Most of the inflammatory mediators are medium sized with medium molecular weight and can easily pass through the AN69-ST filter. IL-6, IL-10, and TNF-alpha have medium to high molecular weight and are cleared by the adsorption method across the membrane of the hemofilter AN69-ST. R. Bellomo et al. and Yekebas et al. in their respective studies found that CVVH can efficiently remove cytokines, TNF, KININ, and phospholipases and decrease the mortality rate for SAP [37, 38]. The FDA approved the use of CVVH in the mid-1980s as an alternative to continuous arteriovenous hemodialysis (CAVHD) [39, 40]. Blinzler et al. in 1991 used CVVH for the first time to treat SAP in the early stage [9].

The pseudocyst is formed due to hypercytokinemia causing injury to the parenchyma or ductal system. CVVH can clear excessive enzymes and serum cytokine levels and improve endothelial and immune function timely, thus preventing pseudocyst formation. During SAP, gut barriers are compromised, leading to translocation of bacteria [41–44], which can result in winning formation. CVVH was carried out with the AN69-ST hemofilter with polyacrylonitrile membrane and polymyxin B column; the AN69-ST hemofilter can clear the endotoxin adsorption process. The adsorption method removes bacterial LPS by charge or hydrophobic interactions due to the presence of lipid A groups. CVVH can effectively improve splanchnic circulation, remove endotoxins, and decrease the bacterial load [45], thus preventing WON formation.

Organ failure generally occurs within the first seventy-two hours after onset; this early stage of organ failure is due to hypercytokinemia [46]. Controlling inflammatory cytokines within 72 hours of onset prevent further organ damage. 48-72 hours of onset is the widely accepted therapeutic window [47, 48]. In the present study, CVVH was started within 2 hours of hospital admission or within 24 hours of onset. In the early stage of SAP, less amount of proinflammatory and anti-inflammatory factors is formed; their molecular weight is less compared to a later stage [49, 50], so early CVVH can easily filter these cytokines. Early removal of cytokines can maintain the microcirculation and oxygen supply to the organs and prevent the progression of tissue damage. Three separate studies in 2000, 2001, and 2009 demonstrated that early CVVH can improve the overall organ function in SAP [51–53].

## 7. Conclusion

Early continuous veno-venous haemofiltration (CVVH) can efficiently balance between proinflammatory cytokines and anti-inflammatory cytokines, which causes leakage of pancreatic enzymes from the pancreatic duct if its persistence will eventually develop fibrosis around its periphery leading to pseudocysts and WOPN. CVVH can efficiently eliminate excess cytokines and enzymes, prevent diffuse inflammatory reactions, and improve endothelial function, intestinal permeability, immune function, vitals, severity, acid-base balance, healing of the inflammation, and immunodepression. CVVH may effectively treat SAP and acute complications (MODS, SIRS, DIC, etc.) of SAP and prevent the formation of acute peripancreatic fluid collection, pancreatic pseudocyst, acute necrotic collection, and walled-off necrosis (WON).

## Figures and Tables

**Table 1:** Modified Marshall scoring system for organ dysfunction (a score of 2 or more in any system defines the presence of organ failure).

Organ system	Score 0	Score 1	Score 2	Score 3	Score 4
Respiratory (PaO_2_/FiO_2_)	>400	301-400	201-300	101-200	≤101
Renal (serum creatinine, mg/dL)	<1.4	1.4-1.8	1.9-3.6	3.6-4.9	>4.9
Cardiovascular (systolic blood pressure, mmHg)	>90	<90 (fluid responsive)	<90 (not fluid responsive)	<90 (pH < 7.3)	<90 (pH < 7.2)

**Table 2:** Comparing baseline at the time of admission.

S.N	Parameters	CVVH (*N* = 18) (pretreatment)	Conventional therapy (*N* = 24) (pretreatment)	*p* value
1	Age	51.77 ± 12.02	53.50 ± 12.89	>0.05
2	Sex (M : F)	13 : 5	17 : 7	NS
3	Amylase	635.55 ± 138.50	632.95 ± 145.14	0.953
4	Lipase	1067.22 ± 153.19	1066.91 ± 124.34	0.995
5	CRP	269.27 ± 75.77	268.79 ± 48.50	0.981
6	IL-6	662.22 ± 166.43	663.25 ± 167.32	0.984
7	IL-10	1104.05 ± 141.03	1105.58 ± 107.14	0.970
8	TNF-alpha	577.94 ± 104.03	578.95 ± 82.95	0.973
9	Ranson score	6.00 ± 1.45	5.79 ± 1.50	0.653
10	APACHE-II score	21.83 ± 1.75	22.66 ± 1.78	0.139
11	Balthazar score	7.27 ± 0.75	6.95 ± 0.75	0.181

**Table 3:** Comparing different parameters in the conventional treatment group pre- and post treatment.

S.N	Parameters	Pretreatment	Post treatment	*p* value
1	Amylase	632.95 ± 145.14	507.87 ± 99.87	0.005
2	Lipase	1066.91 ± 124.34	939.37 ± 118.01	0.002
3	CRP	268.79 ± 48.50	226.50 ± 39.89	0.007
4	IL-6	663.25 ± 167.32	570.12 ± 91.03	0.020
5	IL-10	1105.58 ± 107.14	1024.62 ± 121.87	0.040
6	TNF-alpha	578.95 ± 82.95	517.00 ± 80.05	0.020
7	Ranson score	5.79 ± 1.50	5.00 ± 1.17	0.046
8	APACHE-II score	22.66 ± 1.78	19.45 ± 1.31	<0.01
9	Balthazar score	6.95 ± 0.75	6.62 ± 0.64	0.148

**Table 4:** Comparing different parameters in the CVVH treatment group pre- and post treatment.

S.N	Parameters	Pretreatment	Post treatment	*p* value
1	Amylase	635.55 ± 138.50	242.22 ± 58.85	<0.01
2	Lipase	1067.22 ± 153.19	430.27 ± 98.30	<0.01
3	CRP	269.27 ± 75.77	159.27 ± 16.06	<0.01
4	IL-6	662.22 ± 166.43	274.27 ± 66.34	<0.01
5	IL-10	1104.05 ± 141.03	531.16 ± 76.33	<0.01
6	TNF-alpha	577.94 ± 104.03	307.16 ± 58.78	<0.01
7	Ranson score	6.00 ± 1.45	3.72 ± 0.75	<0.01
8	APACHE-II score	21.83 ± 1.75	11.27 ± 2.05	<0.01
9	Balthazar score	7.27 ± 0.75	3.83 ± 0.85	<0.01

## Data Availability

All data generated or analyzed during this study are included in this article. Further information is available from the corresponding authors on reasonable request.

## Abbreviations

APACHE: Acute Physiology and Chronic Health Evaluation
CRP: C-reactive protein
CVVH: Continuous veno-venous haemofiltration
ICU: Intensive care unit
MODS: Multiple organ dysfunction syndrome
SAP: Severe acute pancreatitis
IL: Interleukin
SIRS: Systemic inflammatory response syndrome
CRRT: Continuous renal replacement therapy
TNF-*α*: Tumor necrosis factor alpha
ARDS: Acute respiratory distress syndrome
APFC: Acute peripancreatic fluid collection
ANC: Acute necrotic collection
WON: Walled-off necrosis.
